# Contrasting internally and externally generated Atlantic Multidecadal Variability and the role for AMOC in CMIP6 historical simulations

**DOI:** 10.1098/rsta.2022.0194

**Published:** 2023-12-11

**Authors:** Jon Robson, Rowan Sutton, Matthew B. Menary, Michael W. K. Lai

**Affiliations:** ^1^ Department of Meteorology, National Centre for Atmospheric Science, University of Reading, Reading, UK; ^2^ Met Office Hadley Centre, Exeter, UK

**Keywords:** Atlantic Ocean, multidecadal variability, AMV, AMOC

## Abstract

Atlantic multidecadal variability (AMV) has long been thought to be an expression of low-frequency variability in the Atlantic Meridional Overturning Circulation (AMOC). However, alternative hypotheses have been forwarded, including that AMV is primarily externally forced. Here, we review the current state of play by assessing historical simulations made for the sixth coupled model intercomparison project (CMIP6). Overall, the importance of external forcing is sensitive to the type of AMV index used, due to the importance of globally coherent externally forced signals in the models. There are also significant contrasts between the processes that drive internally and externally forced AMV, but these processes can be isolated by exploring the multivariate expression of AMV. Specifically, internal variability in CMIP6 models is consistent with an important role of ocean circulation and AMOC and the externally forced AMV is largely a surface-flux forced mechanism with little role for the ocean. Overall, the internal multivariate fingerprint of AMV is similar to the observed, but the externally forced fingerprint appears inconsistent with observations. Therefore, climate models still suggest a key role for ocean dynamics, and specifically AMOC, in observed AMV. Nevertheless, models remain deficient in a number of areas, and a stronger role for externally forced dynamical changes cannot be ruled out.

This article is part of a discussion meeting issue ‘Atlantic overturning: new observations and challenges’.

## 1. Introduction

Over the observed period (circa 1850–today), North Atlantic sea surface temperatures (SSTs) have varied significantly on decadal-to-multidecadal timescales—a phenomenon that has become known as Atlantic Multidecadal Variability (AMV, [1,2]). Importantly, these basin-scale warm or cool anomalies have evolved somewhat differently to global mean temperatures, suggesting that they are caused by different mechanisms. Furthermore, evidence from paleo-proxies suggests that significant decadal timescale variability has existed in the North Atlantic climate system for thousands of years, indicating that AMV is a natural feature of the climate system [3,4].

AMV has been linked to a wide range of important climate impacts. For example, AMV has been associated with rainfall and temperature anomalies over North America [5,6], Europe [7,8] and the tropics including changes in the global monsoon [9,10]. AMV has also been associated with large-scale changes in atmospheric circulation, including the North Atlantic Oscillation (NAO) and the North Atlantic jet in winter [11–13] and summer [14,15]. In addition, AMV has been implicated in driving Pacific decadal variability via its impact on the Walker circulation [16,17], and may have played a key role in shaping the so-called hiatus in global warming in the early 21st Century through such a mechanism [18]. AMV has also been linked to the multidecadal changes in high impact weather, such as the number of hurricanes [9,19] and droughts [6,9,19], changes in fisheries [20,21], and the rate of coastal melt from the Greenland Ice sheet [22]. AMV has also been suggested to modulate global-mean surface temperatures more generally [23], but this link is debated [24].

Although the literature has largely focused on SST anomalies, the observed AMV is a multivariate phenomenon [19,25–27]. For example, the observed AMV SST anomalies are correlated with large changes in upper ocean heat content in the North Atlantic as well as changes in both sea surface salinity and upper ocean salinity, especially over the subpolar North Atlantic (SPNA; [19,26,28,29]). Observations also suggest that AMV is associated with significant changes in surface heat fluxes—i.e. a warm (or positive) phase of AMV is associated with increased heat release from the extra-tropical North Atlantic Ocean and SPNA [30,31]. Many studies have also consistently shown considerable changes in North Atlantic ocean circulation using proxies or indirect observations [1,32–35] that have varied in phase with AMV.

### (a) The internal processes that shape AMV

A long-standing hypothesis in the literature for the existence of AMV is that it is an expression of low-frequency variability in the ocean circulation, and particularly the Atlantic meridional overturning (AMOC) which is a system of ocean currents that moves heat northwards in the North Atlantic [36]. However, due to a lack of direct observations, these conclusions have relied largely on the simulation of AMV in climate models. For example, coupled climate models generally simulate some form of AMV spontaneously in pre-industrial or present-day control simulations, i.e. with no changes in external forcing factors (natural or anthropogenic) [19,37–43]. In other words, AMV can be understood as an emergent phenomenon that arises due to the ‘internal’ interactions between different components of the Earth system. Such mechanisms of AMV usually revolve around changes in the ocean circulation and particularly those associated with the thermohaline or buoyancy forced part of the ocean circulation, i.e. the part that is driven by densification of sea water [19,38,43].

Persistent changes in atmospheric circulation, like the winter NAO, are often thought of as a key driver of such ocean circulation changes [44]. For example, persistent positive NAO, like that observed in the late 1980s and early 1990s, can drive increased heat loss from the SPNA and subsequently strengthen the AMOC [28,45–47]. Indeed, there is evidence to suggest that NAO, AMOC and AMV-like SST variability are coupled [43,48,49]. However, other mechanisms such as the propagation of freshwater from the arctic [39,43,50,51] or salinity anomalies from the south [52] have also been hypothesized as key drivers of AMOC, and hence AMV-like variability. Irrespective of the initial driving mechanisms, the resulting ocean circulation anomalies then drive co-varying changes in both surface temperature and salinity through the impact on ocean heat and salinity transports [19,29]. Increased AMOC would then broadly warm the North Atlantic, which would tend to lower the surface density, which subsequently favours a reduction in the AMOC [41,46]. For a review of the link between AMV and the AMOC, the reader is directed to the comprehensive reviews of Zhang *et al.* [19] and Buckley and Marshall [36].

Although in many models the AMOC has been identified as an important driver of internal AMV, there is an increasing appreciation that other processes are important. For example, changes in ocean mixed layer shape the SPNA SST signal associated with AMV [53]. Changes in atmospheric circulation also drive significant heat fluxes over the North Atlantic [44] and, thus, could feed back onto AMV surface temperatures [43,54]. Indeed, it has been proposed that atmospheric circulation changes, and their impact on local heat fluxes, are the main driver of AMV without a need for ocean circulation responses [55]. However, the idea that ocean circulation is not involved in AMV is heavily debated, as such an interpretation is not consistent with the relationship between AMV and surface heat fluxes [30,31] nor the changes in ocean circulation seen in the models or implied in the observations [1,29,56]. Changes in atmospheric circulation can also bring about changes in cloud cover [54,57,58] and are hypothesized to modulate natural aerosols in the tropics through the impact of AMV-related rainfall and wind anomalies on Sahel dust [59], which can influence SST though their impacts on downwelling shortwave radiation.

Whilst coupled climate models simulate some form of AMV spontaneously in pre-industrial simulations, it is also important to recognize that this simulated internal AMV is unable to recreate various features of the observations. For example, the AMV in models is generally too weak (e.g. it has a smaller magnitude) and shorter in timescale (as measured by autocorrelations) when compared to the observed AMV [41,60]. The spatial pattern of AMV can also be diverse between models [41,42,54]. In particular, the size of the tropical SSTs associated with the simulated AMV is generally smaller than in observations, but there is a significant spread between models [54,60,61].

### (b) The role of external forcings in driving AMV

Given the limitations of the simulated internal AMV to explain the observations, there has been an increasing focus on the potential role of both natural and anthropogenic external forcings. For example, both the total variance and timescale of AMV (e.g. the auto-correlation) increases in simulations that include estimates of past external forcing [62–64]. Studies have shown that natural forcing (e.g. volcanic or solar activity) can project onto and modulate North Atlantic atmosphere and ocean circulation, which in turn can drive AMV-like variability [65–67]. For example, natural forcing can project onto equator-to-pole lower-stratospheric temperature gradients and, hence, drive changes in atmospheric circulation that subsequently drive the AMOC [65]. Alternatively, natural forcings could drive the ocean circulation more directly by altering the heat fluxes or freshwater fluxes over regions in the North Atlantic where the AMOC is sensitive [67,68].

Over the past decade there has also been considerable focus on the role of anthropogenic forcing in driving North Atlantic variability, which raises the possibility that the observed AMV in the latter half of the 20th century was largely a human influenced, rather than a purely natural, phenomenon. For example, many studies have argued that multidecadal timescale changes in anthropogenic aerosol (AA) precursor emissions, particularly over the latter half of the 20th century, contributed significantly to AMV variability by modulating the surface heat budget [62,63,69–72]. Specifically, increases in the emissions of sulphate aerosol precursors from North America and Europe in the 1950s and 1960s led to a decrease in downwelling solar radiation largely through cloud–aerosol interactions, and subsequent cooling of the North Atlantic SSTs and upper ocean [62,63,69,70]. Furthermore, the decline in aerosol precursor emissions from these regions in the 1980s and 1990s led to increased downwelling solar and basin-scale warming of SST [62,63]. Aerosols have also been shown to influence both the atmospheric circulation of the North Atlantic [73–75] and the strength of the ocean circulation including the AMOC in coupled models [75–79], which raises the question of whether anthropogenic forcing may have influenced AMV through these processes too.

### (c) Uncertainties and questions

Although there is emerging evidence that AAs have played an important role in shaping the recent Atlantic variability, there remain a number of inconsistencies with the real world, and so questions remain. For example, many studies have shown that the externally forced AMV SST pattern has significant differences to the observed, particularly in the SPNA [80,81]. Furthermore, the mechanisms that are usually invoked to explain the impact of AA forcing on the North Atlantic SST assume AA impacts AMV only through changes in the surface heat budget (i.e. no role for the ocean circulation) [62,63,69,70]. However, this is inconsistent with the observed multivariate finger print of observed AMV which implicates a role for ocean circulation change [19,26,82,83]. Importantly, there are still large uncertainties in both the strength and regional evolution of the historical aerosol forcing [84,85] and also in how models respond to that forcing. Indeed, it has been shown that AA forcing can lead to diverse impacts on the North Atlantic in models [78,79], and there is emerging evidence that the surface temperature response to aerosols is too large in many models [79,86–88]. Therefore, questions are raised about whether models adequately simulate the impact of aerosols on the North Atlantic.

An additional uncertainty in understanding AMV is the use of different AMV indices in the literature. For example, a common metric is the linearly detrended basin-mean (e.g. $70\text{--}0^{\circ }\;W$, $0\text{--}60^{\circ }\;N$) SSTs, which has been used widely (e.g. [2,5,64,89]). Alternatively, some studies just use the basin-mean SST without detrending (e.g. [62]). However, both indices do not discriminate between variability that is unique to the North Atlantic and global scale variability [19,90]. Other AMV indices have been proposed to address this specific issue, for example by either removing the global-mean temperature anomalies from the basin-mean AMV index [1,90] or by removing the globally forced signal spatially by regressing on the global mean [80,91,92]. More complex indices have also been proposed to address this problem [42]. Therefore, it is important to assess the impact of the choice of AMV index on conclusions that we can draw on the relative importance of internal versus external AMV mechanisms.

It follows that there is significant uncertainty in the importance of different processes in shaping the evolution of the observed AMV. However, given the considerable evidence that the observed AMV has had an important role in shaping regional climate, including high impact weather, there is a pressing need to be able to predict the future evolution of the AMV. Indeed, large changes in AMV could act to exacerbate, or weaken, the expected regional changes in climate from anthropogenic climate change over the next decades (e.g. [93]). Unfortunately, we do not know whether the AMV over the observed time period has been normal or remarkable, and we are unable to confidently predict how we expect AMV to evolve in the coming decades.

Therefore, in this study, we revisit the simulations of AMV in state-of-the-art coupled models with the aim of reviewing and assessing the role of different processes in observed AMV. In particular, we will do this by characterizing, and contrasting, the simulation of both internal and externally forced AMV. Previous work has touched on these issues [26,80], but a systematic analysis has not been applied rigorously across the latest models.

## 2. Methods

### (a) CMIP6 historical simulations and data

We explore the simulation of AMV in the latest coupled model simulations made for phase six of the coupled model intercomparison project (CMIP6 [94]). Specifically, we make use of the historical simulations that cover the period 1850–2014. These historical simulations include time-varying external forcings including greenhouse gas concentrations, natural and anthropogenic aerosols and changes in solar insolation. We use 17 models in total and use up to the first nine members to ensure that multi-model mean (MMM) results are not dominated by a large number of members from one or two models. These models were chosen as they include the variables that are needed for every ensemble member, and are similar to the models used in the study by Robson *et al.* [79] (henceforth, R22), but they also span a range of AMOC mean states and sensitivity to AAs (see table S1, electronic supplementary material, for an overview of the models used).

As in R22, we focus on the MMM results, but we also separate models into two groups that represent their relative sensitivity to historical AAs. We do this by stratifying models according to their simulated trend in interhemispheric top of atmosphere imbalance in absorbed solar radiation (which we called ASR_HD). We stratify models according to whether the linear change in ASR_HD is larger than $1.5\;{W\;m}^{-2}$ over 1850–1985, which is the time period over which AA forcing dominates this metric [78]. Models with a linear change larger than $1.5\;{W\;m}^{-2}$ are labelled as *strong* models, whereas those with a smaller change are labelled as *weak* models. This gives a total of nine strong models and eight weak models. The reader is directed to Menary *et al.* and R22 for further description of the ASR_HD metric.

In this study, we focus exclusively on annual mean data. Surface heat fluxes are defined as positive into the ocean. As in the study by Menary *et al.*, we compute the AMOC time series from the ocean velocity data to be consistent across models. The observed AMV is computed using ERSST version 5 data [95]. Note that there will be uncertainty in observed AMV due to observational uncertainty, but previous studies have indicated that the difference in the statistics of AMV (e.g. the variance) are less sensitive to specific data set compared to the index used [60]

### (b) AMV indices and decomposing the AMV into internal and external components

We will compute and compare the results from two AMV time series in this article. The first AMV index is the widely used linearly detrended basin-mean Atlantic SSTs, which we call **AMV_BM**. The index is computed by first making an area weighted average of SST over the region $0\text{--}65^{\circ }\;N$ and $0\text{--}75^{\circ }\;W$. The resulting time series is then linearly detrended over 1850–2014 to remove an estimate of the globally forced signal.

Although the AMV_BM index is widely used, it is known to have limitations as previously mentioned, not least because global signals due to greenhouse gases and AA forcing do not evolve linearly through time [19,90]. Thus, we also utilize the index used in Andrews *et al.* [80], which we will call **AMV-Glob**. That is, SST anomalies associated with global-mean changes are first removed by linearly regressing the annual mean time series at each grid point onto a smoothed time series of global-mean ($60^{\circ }\;S\text{--}60^{\circ }\;N$) SST, which we smooth with a 10-year running mean. Hence, this method removes a temporally nonlinear estimate of global SST signals. The resulting SSTs are then averaged over the North Atlantic basin (e.g. $0\text{--}65^{\circ }\;N$ and $0{\text{--75}}^{\circ }\;W$). All AMV indices are subsequently smoothed by a 10-year rolling mean. Note that due to the use of 10-year smoothed global-mean temperatures to remove long-term globally forced changes from the AMV-Glob, this restricts the available years to 1860–2009 in CMIP6 historical runs, and comparison with the observations is done over this period for both AMV indices.

In CMIP6 historical simulations, we assume that we can decompose the total AMV into internal and external components, which we will call iAMV and fAMV. In particular, we assume that, for each model separately, the total AMV is the sum of the iAMV and fAMV (i.e. AMV=iAMV+fAMV). By exploiting the fact that making an ensemble mean should remove the internal variability to reveal the common forced response, we first compute fAMV for each model individually by using the ensemble mean SST. To compute the iAMV, we compute the relevant AMV index from each member separately, and then make this index relative to the fAMV index for that model; in other words, the iAMV for each individual member is defined as the residual variability (e.g. $iAMV_{n}^{m}=AMV_{n}^{m}-fAMV^{m}$, where n is the ensemble member and m is the model). This method of separating externally forced and internal (or residual) variability has been used previously [92,96] and is often referred to as ‘de-meaning’ the ensemble. Note that, when characterizing the iAMV relationships for each model, we compute the desired metric for each member separately and then take the ensemble mean of the metric. When computing the AMV-Glob index for each ensemble member, we always use the model’s ensemble mean global-mean SST to estimate, and remove, the externally forced temperature changes. For the iAMV_BM, we compute the linear trend from the ensemble mean. Broadly we find that this method is able to isolate similar magnitude variability to that simulated in pre-industrial control simulations (i.e. the internal variability), although when applying AMV-Glob to a single index can suppress AMV variability in models where the internal AMV has a large global expression (see figure S1, electronic supplementary material). Note that we also use the same method to split AMOC variability into its externally forced (fAMOC) and residual (i.e. internal, iAMOC) variability.

## 3. Results

### (a) Characterizing the observed AMV and the sensitivity to AMV index

Before exploring the AMV in coupled simulations, we first turn our attention to the observations to highlight the sensitivity of AMV to the index used.

**Figure 1.**
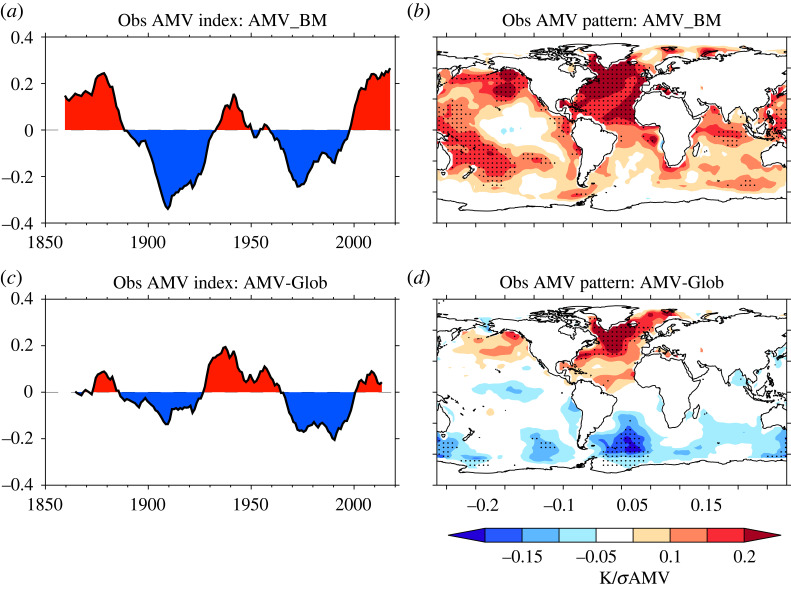
Compares the observed AMV time series and AMV pattern for the two definitions of AMV used in this study. (*a*) The AMV_BM index. (*b*) The resulting AMV pattern computed as a regression of SST anomalies onto the standardized AMV_BM index at each grid point. Stippling shows where the regression slope parameter is significant at the p≤0.1 based on a block-bootstrap re-sampling of the AMV index. Blocks were 10 years long, and we used 500 Monte Carlo permutations. (*c*) and (*d*) show the same as (*a*) and (*b*) but now for the AMV-Glob index. See §2(b) for details on the indices used. Regression patterns are computed over the same period 1860–2009.

Overall, although we find that there is some sensitivity to the definition of the AMV index, some key features of the observed AMV are broadly consistent across the two AMV indices used. For example, figure 1 shows that the phases of AMV variability are similar between the AMV_BM (figure 1*a*) and AMV-Glob index (figure 1*c*). Furthermore, there is broad consistency in terms of the pattern of SSTs associated with AMV in the North Atlantic (i.e. the AMV pattern). In particular, positive AMV is associated with a widespread warming across the North Atlantic region, but the warming is largest in the SPNA and with warm anomalies also present in the tropical North Atlantic (compare figure 1*b*,*d*).

Nevertheless, there are some differences in the properties of the observed AMV when using the two different indices. In particular, the magnitude of the AMV-Glob index is smaller than that for the AMV_BM; specifically, the variance of the 10-year smoothed index is 0.025 K and 0.010 K in the AMV_BM and AMV-Glob, respectively. The difference in variance is largely due to differences in the magnitude of the AMV anomalies prior to 1930, but also after 2000. There are also some slight changes in the timing of the AMV anomalies between the two, particularly related to the mid-20th century transition from positive to negative AMV anomalies, which occurs in the mid-1950s in AMV_BM, but in the 1960s in AMV-Glob. In terms of the AMV pattern, AMV_BM appears to be related more strongly to the Pacific, with a warming over the western Pacific and less warming over the eastern Pacific reminiscent of the spatial pattern of the Interdecadal Pacific Oscillation [17]. The AMV-Glob has a much weaker relation to the Pacific in contrast to the results of [92] who show that the AMV-Glob can erroneously find a relationship with the Pacific. However, [92] only uses data from 1950 onwards, which furthermore underlines the sensitivity of results to the choice of AMV index.

### (b) Characterizing the importance of externally forced AMV variability and the sensitivity to the AMV index

**Figure 2.**
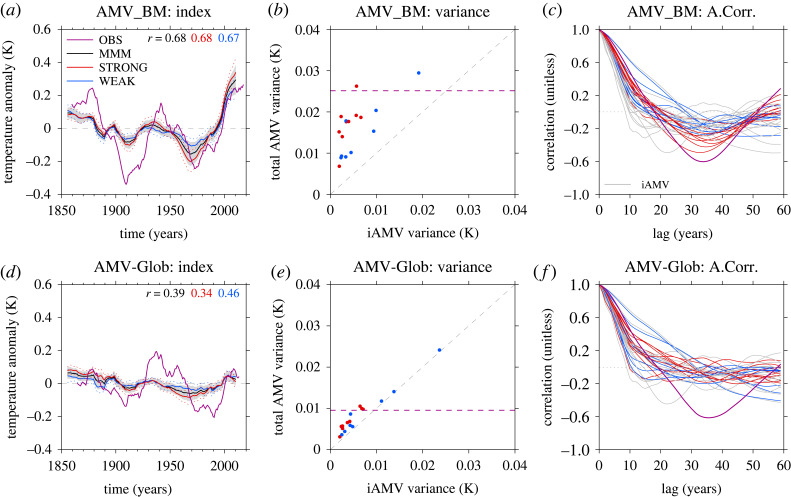
The sensitivity of the temporal variability of the simulated fAMV to the AMV index used. (*a*) The fAMV_BM index for the MMM (black) compared to the observed AMV_BM index (purple). Also shown is the fAMV_BM index for the strong (red) and weak (blue) sub-ensembles. Black shading shows the 1σ spread of the MMM ensemble mean, and red and blue dots show the spread of the strong and weak, respectively. Numbers in top of the panel show the temporal correlation between the simulated ensemble mean time series and observations. (*b*) The comparison of variance of the iAMV and total AMV for AMV_BM as a scatter plot. Each model is represented by a red or blue dot (to signify the models that make up the strong or weak sub-ensembles), and shows the ensemble mean iAMV variance (x-axis) compared to the ensemble mean variance of the total AMV (y-axis). The purple horizontal line shows the total variance for AMV_BM computed from observations, and grey dashed line shows the one-to-one line. (*c*) The ensemble mean autocorrelation for iAMV (grey) and fAMV (red and blue) in each model for AMV_BM. Purple shows the observed autocorrelation. (*d*–*f*) The same as (*a*–*c*) but now for the AMV-Glob index.

**Figure 3.**
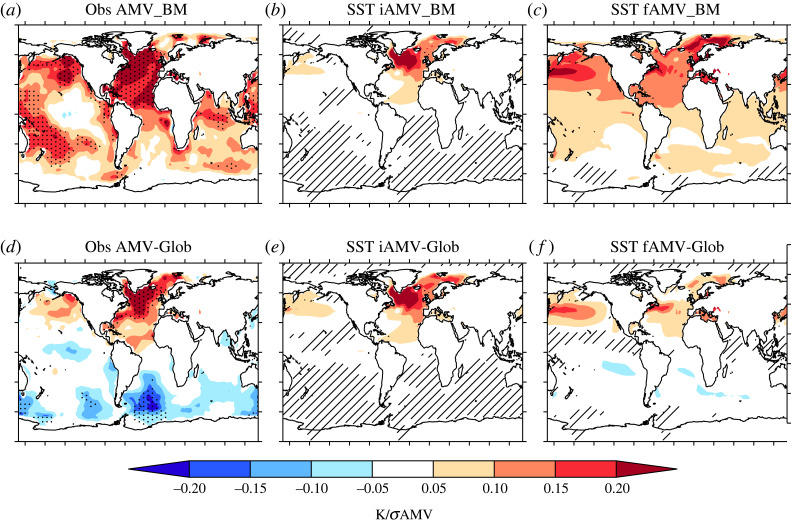
Comparison of the AMV pattern between internal and external components of AMV and with that computed from observations. (*a*) The AMV pattern for observations computed using the AMV_BM index after it has been normalized by its standard deviation (K/σAMV) (note this is the same as shown in figure 1). (*b*) The same as (*a*) but now for the average of all iAMV patterns. Hatching shows where 80% of models do not agree on the sign of the regression slope coefficient. (*c*) The same as (*b*) but now for the fAMV. (*d*–*f*) The same as (*a*–*c*), but now for the AMV-Glob.

We now turn our attention to the importance of AMV index in CMIP6 historical simulations, particularly in its impacts on the conclusions of how important external forcing is in driving AMV. In particular, figure 2 shows a comparison of the simulated fAMV index with observations and figure 3 shows the comparison of the simulated AMV patterns.

When using the AMV_BM index, there appears to be considerable temporal agreement between the MMM AMV_BM index and that observed in terms of phasing of AMV. For example, the MMM fAMV_BM shows negative values in the early ∼1900s--1920s and ∼1950s--1980s and positive values in the mid-20th century and after the year 2000. Indeed, the temporal correlation between the MMM fAMV_BM index and observed AMV_BM is 0.68 (figure 2*a*). The agreement between the simulated fAMV_BM is particularly strong after ∼1950 where the magnitude of the simulated anomalies in the MMM fAMV_BM index is similar to the observed AMV_BM index.

In addition to the temporal agreement, using AMV_BM suggests external forcings have a substantial impact on both the total magnitude of AMV and the persistence/timescale of AMV variability. For example, figure 2*b* shows that the *total AMV variance is substantially larger than iAMV_BM in every model*. Specifically, the magnitude of the total AMV_BM variability in each model is shifted above the 1-to-1 line and the variance of the MMM AMV_BM, which is 0.125 K, is ∼2 times larger than the MMM of the iAMV_BM simulated across models. Furthermore, figure 2*c* shows that the time scale of AMV variability is also larger for the fAMV_BM compared to the iAMV_BM in many models, with some models even exhibiting a minimum in autocorrelations at just over 30 years consistent with that observed. Nevertheless, it is important to note that most of the models still simulate weaker AMV variability than observed (e.g. figure 2*b*), and all models still underestimate the negative autocorrelations seen in the observed AMV_BM (e.g. figure 2*c*).

In contrast, the apparent impact of external forcings is reduced when using the AMV-Glob index. For example, the temporal agreement with the simulated MMM fAMV-Glob is substantially weaker; the temporal correlation between the MMM fAMV-Glob and the observed AMV-Glob is only 0.39 (figure 2*d*). Furthermore, the impact of external forcing on the magnitude and persistence of the simulated AMV is also substantially smaller. For example, in terms of comparing the total vs iAMV variance, the models generally lie close to the 1-to-1 line when using AMV-Glob. For the MMM, the total variance of AMV-Glob is 0.088 K and is only 25% larger than the MMM variance of iAMV-Glob (figure 2*e*). Therefore, *although there is undoubtedly a forced signal in AMV-Glob in CMIP6 historical simulations, it is small compared to the internal variability*. There are also only small changes in the autocorrelation of AMV-Glob between fAMV-Glob and iAMV-Glob, and few models have a negative autocorrelation values similar to that in observations (figure 2*f*).

The apparent importance of the fAMV is also sensitive to whether the models fall into the strong or weak category. In terms of the magnitude of fAMV variability, we find that the strong models have stronger cooling after 1950 than the weak models in both indices, which results in stronger changes in the total variance of AMV. For example, when using the AMV_BM, we find that the standard deviation of the total AMV is approximately 2.3 times larger than for iAMV_BM in the strong models, but only 1.7 times larger for the weak (e.g. figure 2*b*,*e*). Furthermore, this difference in the relative increase in total AMV standard deviation between strong and weak models is statistically significant at the p≤0.05 level based on a Student’s *t*-test. The models that show the most similarity with observations in terms of autocorrelations are also all strong (e.g. figure 2*c*). In contrast, the changes in the variance for the AMV-Glob are smaller, but still dominated by the strong models. The total AMV standard deviation in the strong models is 1.3 times larger than internal variability, which is also statistically larger than the 1.1 times more variance in the weak models at the p≤0.05 level.

The sensitivity of the fAMV to the index used and, hence, the conclusions on the importance of fAMV appears related to the presence of hemispherically coherent externally forced signals in surface temperature. Indeed, the spatial pattern of SST anomalies associated with the fAMV_BM is a broad temperature increase across the majority of the Northern Hemisphere (figure 3*c*), consistent with [80] and [61]. For the fAMV-Glob, the broad positive relationship across the Northern Hemisphere has reduced somewhat, but there is still a substantial warming across the Northern Pacific, and the interhemispheric nature of the response is also evidenced by consistent, but weak, cool anomalies over the southern hemisphere (figure 3*f*).

Although there are some similarities of the SST pattern associated with fAMV and the observed AMV pattern, it is important to note that there are a number of striking differences in the North Atlantic and between fAMV and iAMV. In particular, *the fAMV_BM SST pattern is focused on the subtropical North Atlantic rather than the SPNA*. Indeed, for fAMV-Glob, there is little signal in the SPNA across models, consistent with previous analysis [80]. However, note that the SPNA dominates the simulated iAMV and observed AMV for both AMV indices. Another key difference is the SST anomalies in the North Pacific. For example, temperature anomalies in this region appear to be strongly associated with fAMV; this is true for both fAMV_BM and fAMV-Glob indices, but is particularly clear for the fAMV_BM where there is warming across the entire North Pacific. However, in contrast, the simulated iAMV is dominated by SST anomalies which reside largely in the North Atlantic consistently across models for both indices, and the global teleconnections are also weak across models.

In summary, we find that conclusions on the relative contribution of fAMV to the total AMV depend significantly on the AMV index used. In particular, the temporal correlation between simulated fAMV and observed AMV drops substantially when making the North Atlantic relative to global changes (i.e. using AMV-Glob rather than AMV_BM) as does the impact of forcings on the total variance of the AMV and the time scale of AMV (i.e. the autocorrelation). This appears to be due to the fact that the fAMV is strongly related to hemispheric externally forced signals in the models, but the changes in the North Atlantic basin are relatively more important in the observations (and for iAMV). Furthermore, the simulated fAMV is particularly large in the strong in models, which is consistent with the stronger externally forced interhemispheric signals in these models that also appears to be unrealistic [79]. Therefore, due to the sensitivity of the AMV_BM index to hemispherically forced signals, and for brevity, in the rest of this article, we will focus on results from the AMV-Glob. However, the general conclusions on the processes responsible for fAMV and iAMV are not overly sensitive to the index used (see supplementary figures, electronic supplementary material).

### (c) Surface-flux drivers of externally forced AMV

**Figure 4.**
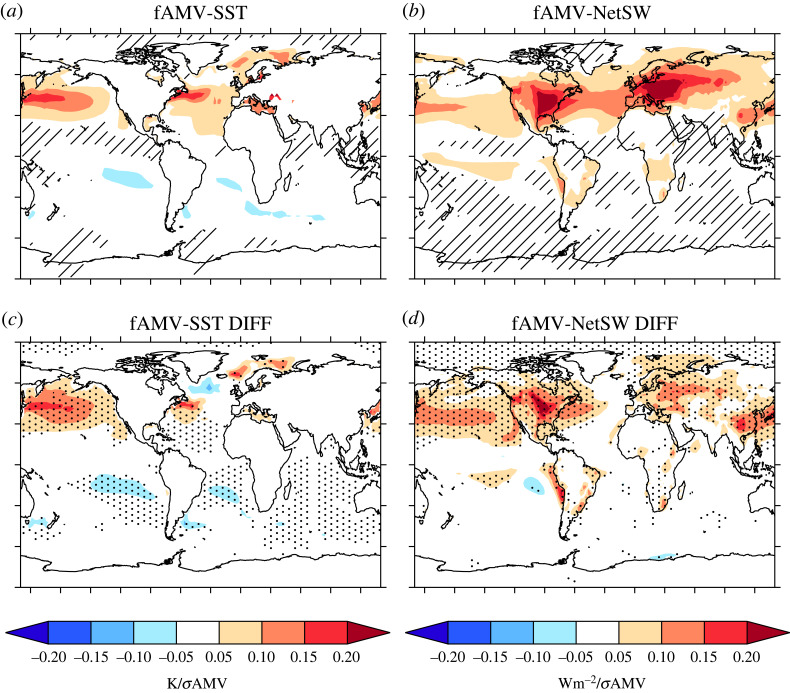
The relationship between the fAMV-Glob and surface heat fluxes. (*a*) The MMM fAMV-Glob SST pattern (note this is the same as shown in figure 3). Contours show the MMM regression pattern of SST on fAMV-Glob, and hatching shows where 80% of models do not agree on the sign of the regression slope coefficient. (*b*) The same as shown in (*a*) but now for net surface shortwave (NetSW). (*c*) The difference in the SST fAMV pattern between strong and weak models (e.g. strong *minus* weak), and stippling now shows where the difference is significant at the p≤0.05. (*d*) The same as shown in (*c*) but for the NetSW.

We now turn our attention to the drivers of the simulated fAMV. Consistent with previous studies (e.g. [62]), we find that the simulated fAMV is associated with changes in net surface shortwave radiation (NetSW) in CMIP6 historical simulations. Figure 4*b* shows that a warming phase of the fAMV-Glob is associated with increased downwelling shortwave across much of the North Atlantic basin. Furthermore, there is a broad consistency between the spatial pattern of the NetSW changes associated with the simulated fAMV-Glob and the simulated fAMV-Glob SST pattern (figure 4*a*). In particular, the NetSW pattern is dominated by anomalies in a band in the northern subtropical North Atlantic (i.e. $30\text{--}45^{\circ }\;N$, as in [62]) with anomalies also reaching down the coast of Africa in the eastern subtropical North Atlantic towards the tropics.

There are also significant differences between the strong and weak models in terms of their relationships between fAMV-Glob and the patterns of SST and heat fluxes. In terms of SST, strong models have a cooler SPNA, and warmer gulf stream extension, but these differences do not appear significant. However, there is a significant difference in terms of the fAMV-Glob relationship with the North Pacific SSTs; in other words, the hemispheric nature of fAMV is more a feature of the modes with strong aerosol forcing/response than those models with weak forcing/response. Furthermore, this difference in the hemispheric nature of fAMV is also consistent with co-incident changes in NetSW, indicating larger changes in NetSW across much of the Northern Hemisphere in the strong models than weak.

### (d) The role of AMOC in AMV

We now explore the role of AMOC in the simulated AMV, which shows substantially different relationships when we consider the internal or externally forced component of AMV.

**Figure 5.**
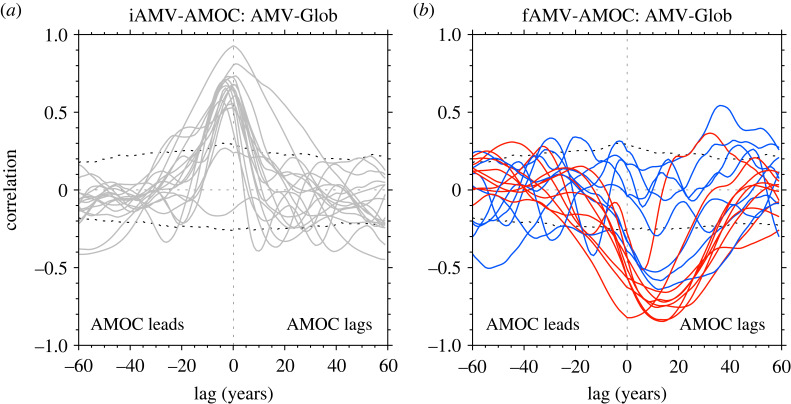
The relationship between AMOC and internal and external components of AMV (e.g. iAMV and fAMV, respectively). (*a*) The cross-correlation between AMOC and iAMV-Glob for each model. Note that the correlations are based on the average of the lagged correlation computed across ensemble members of each model. Negative lags show where AMOC leads iAMV-Glob, and positive lags show where AMOC lags iAMV-Glob. Dotted lines show the 5–95% confidence interval based on a Monty Carlo re-sampling of AMV variability. (*b*) The same, but for correlating each models ensemble mean AMOC with fAMV-Glob for the strong (red) and weak (blue) models.

Overall, AMOC variability plays an important role in the iAMV in CMIP6 models. In particular, figure 5*a* shows the lagged correlations between the AMV-Glob and AMOC for each model. For the iAMV-Glob (figure 5*a*), we find that AMOC at $30^{\circ }\;N$ consistently leads the iAMV by a few years across nearly all models. Furthermore, the iAMV-AMOC relationship is similar to the AMV–AMOC relationship computed from pre-industrial control simulations, underlining that the iAMV decomposition is focusing on mechanisms of internal AMV variability (figure S6, electronic supplementary material, shows the comparison for a sub-sample of the models with comparable data). Nevertheless, most models have a significant correlation of ∼0.6--0.7, indicating that AMOC does not explain all of iAMV variability (consistent with [97]), but some models show a correlation of ∼0.9. Therefore, the relationship between iAMV and AMOC in CMIP6 models is broadly consistent with the hypothesis that AMOC is a key driver of iAMV in coupled models.

In contrast, we find the opposite relationship is true for fAMV in many models (figure 5*b*). Specifically, a decrease in AMOC at 30${\;}^{\circ }$ N is closely associated with a positive fAMV, although the largest negative correlations occur ∼10--15 years after peak AMV in many models. In other words, *the externally forced AMOC is responding to the externally forced changes in the North Atlantic SST anomalies in CMIP6 models, rather than driving them* (consistent with [96] and [70]). Furthermore, the negative correlations between AMOC and fAMV are dominated by the strong models, which are the models with large AA forced changes in AMOC (e.g. R22).

### (e) Contrasting the multivariate nature of AMV between internal and externally forced components

**Figure 6.**
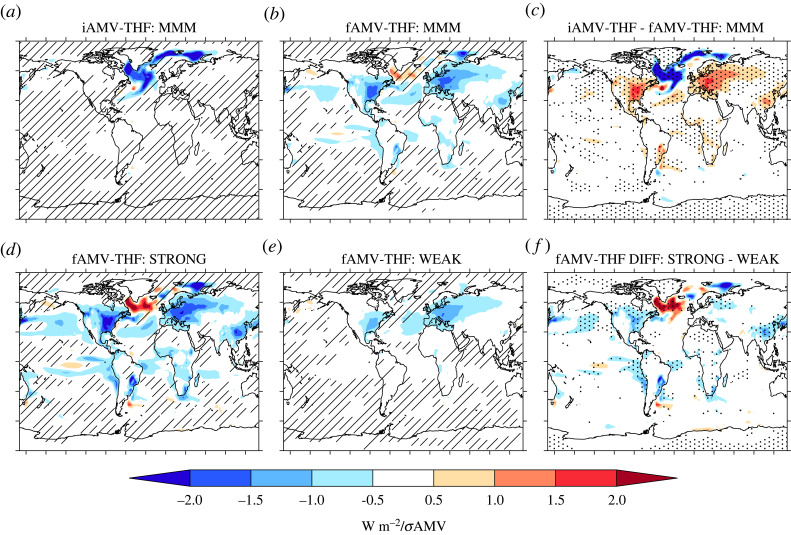
The relationship between AMV and turbulent heat flux (AMV-THF) across internal and external components of AMV. (*a*) The MMM regression between the iAMV-Glob index and turbulent heat flux at lag 0 (computed separately for each member and then averaged). Negative values shows increased turbulent heat flux out of the ocean. Hatching indicates where at least 80% of the models do not agree on the sign of the regression slope. (*b*) The same as (*a*) but for the fAMV-Glob index. (*c*) The difference, i.e. iAMV *minus* fAMV. Stippling shows where the differences in regression slopes are significantly different at the p≤0.05 level. (*d*) and (*e*) The same as (*b*) but now for only models with strong or weak response to AAs (see text). (*f*) The same as (*c*) but now for the strong *minus* weak models.

**Figure 7.**
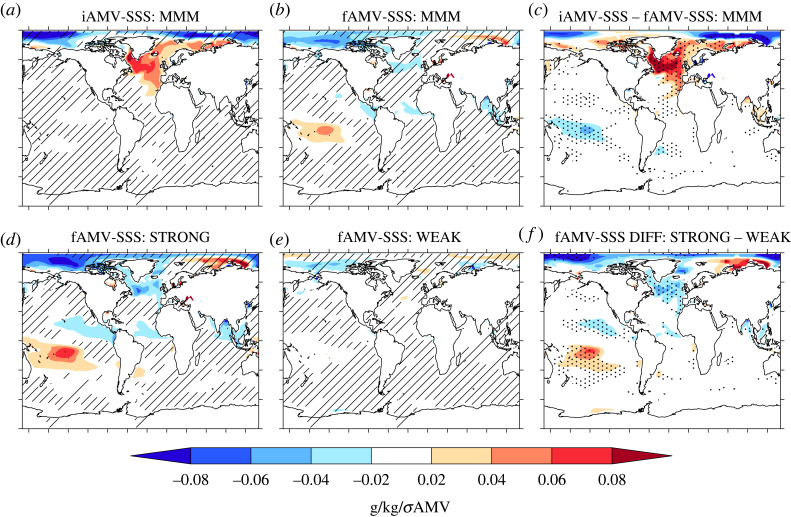
The same as shown in figure 6 but now for the relationship between AMV and sea surface salinity (AMV–SSS) across the internal and external components of AMV.

So far we have largely explored the simulated AMV in terms of surface temperature, and what drives those changes. However, as previously discussed, the AMV is a multivariate phenomenon and, therefore, it is appropriate to examine the simulated relationships of iAMV and fAMV with other variables. In particular, we focus on the relationship between AMV and turbulent heat fluxes (which we call AMV–THF, and show in figure 6) and sea surface salinity (which we call AMV–SSS and show in figure 7).

Over the North Atlantic, we see a contrasting relationship between AMV and turbulent heat fluxes for the internally and externally forced components of AMV. In particular, the MMM iAMV–THF relationship is negative over the SPNA, indicating that THFs are damping iAMV SSTs in the SPNA (figure 6*a*). This damping of SPNA SSTs is broadly consistent with observations [30,31]. Increased heat loss is also seen across the East Greenland current and into the Barents sea. However, the MMM fAMV–THF relationship is opposite, with THF acting to warm much of the SPNA (figure 6*b*). Indeed, the difference between the MMM iAMV–THF and MMM fAMV–THF relationship is statistically significant over much of the SPNA (figure 6*c*). In contrast, the subtropical North Atlantic is dominated by negative fAMV–THF (figure 6*c*), indicating that the atmosphere is acting to damp forced SST anomalies here.

There is also a contrasting relationship between the MMM AMV–SSS for the simulated iAMV and fAMV. In particular, the MMM iAMV–SSS is positive (indicating salinification) over much of the North Atlantic, and particularly the SPNA (figure 7*a*), the Greenland Iceland and Norwegian (GIN) Seas and the Barents Sea. This salinification of the North Atlantic is consistent across models and is also consistent with the expected role of AMOC in iAMV (figure 5*a*; e.g. as stronger AMOC transports more salt northwards). Such a positive relationship is also consistent with the changes observed over the latter part of the 20th century [26,82]. However, in contrast, the MMM fAMV–SSS is negative over the SPNA (indicating a freshening), but it is less consistent across models (figure 7*b*). Nevertheless, the difference in the MMM AMV–SSS relationships is significant over the SPNA and also over the tropics (figure 7*c*). Over the Arctic, there are also large anomalies for both iAMV and fAMV, but these are not consistent across models (similar to [43]) and are not significantly different.

Interestingly, the contrasts between fAMV and iAMV for the THF–AMV and SSS–AMV relationships is largest in the models with strong AA forcing/response. Indeed, the strong models simulate a consistent negative fAMV–THF relationship across the SPNA (figure 6*d*). In contrast, the weak models simulate a more complicated and less consistent relationship with a weaker positive relationship in the eastern SPNA (figure 6*e*). This difference in the fAMV–THF relationship indicates that the atmosphere driven cooling of the SPNA in the MMM (e.g. figure 6*b*) is largely a feature of the strong models (figure 6*d*). Furthermore, the strong models also simulate a negative fAMV–SSS relationship (i.e. freshening) of the SPNA, whereas weak models have a small positive fAMV–SSS in the North Atlantic Current region (figure 7*e*). These differences lead to significant differences between the fAMV–SSS between the strong and weak models across much of the North Atlantic, including a significantly larger freshening in the tropical North Atlantic (figure 7*f*).

## 4. Discussion, recommendations and key questions

In this article, we have used CMIP6 historical simulations and different AMV indices to review the current state of play in the simulation of AMV. Nevertheless, there are still some outstanding issues that need discussing.

It is clear that the choices made in the definition of AMV, e.g. how the index is computed, add to the uncertainties in understanding AMV. Therefore, it is important to reflect this uncertainty when exploring AMV. This could be as simple as using multiple definitions (e.g. as in [60]). Although there is undoubtedly an externally forced component of AMV in CMIP6 historical simulations (e.g. [62,63]), the size of the estimated fAMV is highly sensitive to the choices made in the analysis. This sensitivity appears to be because fAMV in the CMIP6 models is not an Atlantic-only phenomena. Instead, fAMV in CMIP6 is an expression of interhemispheric external forcing that can vary significantly across models (e.g. [79,88]). Therefore, *it is crucial that research into the AMV, and especially the forced component of AMV, not solely be based on the linearly detrended basin-mean AMV index*. When we remove globally coherent forced variability through regression, we find that external forcing only explains a relatively small amount of the total variance, which could be interpreted as that the AMV is primarily internal. However, the method of decomposing the AMV into external and internal components does not take account of nonlinear interactions between external forcing and internal variability. Therefore, the external forcing may still play an important role in phasing the internal variability.

This article underlines that the multivariate nature of AMV is a key aspect of both the observed and simulated AMV (both internally and externally forced AMV). Overall the similarity between the simulated iAMV, e.g. where changes in ocean circulation appear to be important, and the observations supports previous conclusions that there has been an important role for ocean circulation, including the AMOC, in the observed AMV (in agreement with previous studies [19,56,83]). However, in contrast, the simulated fAMV in CMIP6 models appears to be largely a passive-ocean surface-flux forced mechanism that does not appear to explain the observed co-variability of variables. Hence, it is important that the multivariate fingerprint of AMV continues to be used to differentiate between AMV mechanisms, especially where there is an active role for the ocean versus primarily surface-flux forced mechanisms (as previously argued [19,26]). However, to be able to confidently attribute observed variability, we need to ensure that we understand such multivariate relationships in models and in the real world. The results presented here also further underline that different mechanisms dominate in different regions (e.g. subpolar vs subtropics), consistent with previous works [19,43,54,70,98]. Therefore, there *is a need to understand the processes that lead to regional changes in SST, rather than focus on basin-mean changes*. Continued, and improved, long-term observations, including of AMOC and surface fluxes, are necessary to achieve this.

A final challenge is the quality of the models that we use to simulate, and attribute, both the iAMV and fAMV. Indeed, models have significant biases, and this likely affects how the ocean responds to various forcing factors and simulates internal variability (e.g. [99,100]). Therefore, it is important to reflect this uncertainty in analyses of models and not focus on the MMM. In particular, there remains large uncertainty in the magnitude of simulated historical forcings, particularly AA forcing [85]. Indeed, the sensitivity to AA forcing appears to be too strong in many CMIP6 models [79,86–88]. Therefore, improved estimates of historical AA forcing are crucial to make progress as well as improved understanding of how models respond to AA forcing. Another major limitation of coupled models is that they appear to underestimate the influence of both external forcings or internal variability on the atmospheric circulation—the so-called signal-to-noise paradox [101]. Therefore, on the one hand, the external forcing of ocean and atmosphere dynamics associated with AMV by natural or anthropogenic forcings are likely too weak. Consequently, *the question of how important external forcings have been in driving the observed AMV though the modulation of atmosphere and ocean dynamics is still open*. Nevertheless, on the other hand, the internal dynamical mechanisms are also likely underestimated due to the same signal-to-noise issues (e.g. [102]). Therefore, to fully understand and attribute AMV (e.g. to externally forced or internal variability), it is crucial that we continue to improve models. Given the signal-to-noise issues, large ensemble simulations (e.g. [103]) will be important for understanding the robustness of different choices (e.g. [92]) and to pick apart the important simulated mechanisms.

## 5. Conclusion

In this study, we have characterized and explored the simulation of Atlantic multidecadal variability (AMV) in CMIP6 historical simulations. In particular, we have contrasted the internally and externally forced components of AMV, which we term iAMV and fAMV, respectively. The key results are as follows:

— The characteristics of fAMV and, hence, the conclusions on how important external forcing have been in generating the observed AMV are highly sensitive to the AMV index used. In particular, using the linearly detrended basin-mean SSTs suggests external forcings dominated the magnitude and phasing of historical AMV, especially after 1950 in models with a strong sensitivity to AA forcing. However, removing the global-mean forced response via regression on the global mean reduces the apparent importance of the externally forced component of AMV significantly.
— The sensitivity to the AMV index is because the simulated fAMV is part of broad hemispheric-wide externally forced temperature changes. In other words, the fAMV in models is largely a reflection of the forced changes in the Northern Hemisphere, rather than the North Atlantic itself, again especially in models with a strong sensitivity to AA forcing. In contrast, the observed and iAMV signals are more focused on the North Atlantic and are, hence, less sensitive to the removal of the global changes.
— There are significant differences in the SST patterns associated with the fAMV and iAMV. In particular, the fAMV SST pattern is largely associated with mid-latitude SSTs, but the iAMV SST pattern is dominated by subpolar SSTs. The iAMV SST pattern is broadly consistent with that observed, but the majority of models still significantly underestimate the magnitude of the observed variability.
— The iAMV is broadly consistent with an important role for ocean circulation changes. However, in contrast, the fAMV appears to largely be a passive surface–flux forced mechanism (consistent with [62]).
  — For example, iAMV is consistently associated with changes in the strength of the Atlantic Meridional Overturning Circulation (AMOC) leading iAMV. However, the relationship with AMOC and fAMV generally shows a weaker AMOC lagging a warming of SSTs.
  — There is a stark contrast in the multivariate nature of the internal and externally forced AMV. For example, iAMV is consistently associated with an increased turbulent heat release from ocean to atmosphere and a salinification of the SPNA across models. Furthermore, these subpolar relationships for iAMV appear consistent with previous observation-based studies [29–31]. However, fAMV is associated with reduced turbulent heat loss and, in some models, a freshening of the SPNA.

Given the importance of the type of AMV index used for conclusions about externally forced AMV, then it is crucial that future research does not just focus on the basin-mean AMV index. Indeed, given that the changes in global-mean temperatures on decadal timescales are likely to be externally forced, removing some estimate of global-mean temperatures seems to be the most appropriate to isolate the variability specific to the North Atlantic. Once the globally forced signal is removed, the magnitude of the externally forced AMV is small in CMIP6 simulations.

The similarities between the multivariate nature of the iAMV and the relationships found in observations, especially in the SPNA [29–31], suggest that similar processes are in operation in the real world. In other words, CMIP6 models suggest that ocean circulation change has played an important role in shaping the observed AMV. In contrast, a passive-ocean surface-flux forced phenomenon that dominates the externally forced response in CMIP6 models does not appear consistent with observations. Therefore, CMIP6 simulations could be argued to broadly support previous literature that internal changes in ocean circulation, and AMOC, drive the observed AMV.

However, it is important to recognize that there remain significant deficiencies in the simulation of the iAMV. Specifically, iAMV is generally too weak and the timescale of iAMV is too short across most models studied here. Furthermore, there remain significant shortcomings in the current models in terms of biases or errors, in particular, due to the signal-to-noise problems inherent to current coupled models (e.g. [101]). Hence, it is plausible that models incorrectly simulate the dynamical impact of external forcing (e.g. on atmosphere and ocean circulation) and that the externally forced AMV is also deficient in current models. Therefore, due to these model deficiencies and a lack of observational data, it is extremely difficult to estimate the extent to which the AMV has been internally generated or externally forced in the real world.

To make progress, it is crucial that we continue to understand the processes that shape the regional changes in SST in observations and models. In particular, we should avoid only exploring basin-mean SSTs and instead focus on regional multivariate fingerprints of change to reveal the key processes. Furthermore, to attribute the historical AMV and to predict the future, it is important that we continue to improve models, not least to address the signal-to-noise problems. Confronting models with diverse observational based constraints, including high-quality AMOC observations at key locations, and evaluating the relative importance of different mechanisms is crucial to unravel the wider dynamics of the North Atlantic and to improve future predictions.

## Data Availability

All data used within in this study is published on the ESGF as part of CMIP6. Supplementary material is available online [104].

## References

[1] Sutton RT, McCarthy GD, Robson J, Sinha B, Archibald AT, Gray LJ. 2018 Atlantic multidecadal variability and the U.K. ACSIS program. Bull. Am. Meteorol. Soc. **99**, 415-425. (10.1175/BAMS-D-16-0266.1)

[2] Kerr RA. 2000 A North Atlantic climate pacemaker for the centuries. Science **288**, 1984-1985. (10.1126/science.288.5473.1984) 17835110

[3] Chylek P, Folland C, Frankcombe L, Dijkstra H, Lesins G, Dubey M. 2012 Greenland ice core evidence for spatial and temporal variability of the Atlantic Multidecadal Oscillation. Geophys. Res. Lett. **39**, 2012GL051241. (10.1029/2012GL051241)

[4] Wang J, Yang B, Ljungqvist FC, Luterbacher J, Osborn TJ, Briffa KR, Zorita E. 2017 Internal and external forcing of multidecadal Atlantic climate variability over the past 1,200 years. Nat. Geosci. **10**, 512-517. (10.1038/ngeo2962)

[5] Sutton RT, Hodson DLR. 2005 Atlantic ocean forcing of north american and european summer climate. Science **309**, 115-118. (10.1126/science.1109496) 15994552

[6] Ruprich-Robert Y, Delworth T, Msadek R, Castruccio F, Yeager S, Danabasoglu G. 2018 Impacts of the atlantic multidecadal variability on north american summer climate and heat waves. J. Climate **31**, 3679-3700. (10.1175/JCLI-D-17-0270.1)

[7] Sutton RT, Dong B. 2012 Atlantic Ocean influence on a shift in European climate in the 1990s. Nat. Geosci. **5**, 788-792. (10.1038/ngeo1595)

[8] Qasmi S, Sanchez-Gomez E, Ruprich-Robert Y, Boé J, Cassou C. 2021 Modulation of the occurrence of heatwaves over the Euro-Mediterranean region by the intensity of the Atlantic multidecadal variability. J. Climate **34**, 1099-1114. (10.1175/JCLI-D-19-0982.1)

[9] Zhang R, Delworth TL. 2006 Impact of Atlantic multidecadal oscillations on India/Sahel rainfall and Atlantic hurricanes. Geophys. Res. Lett. **33**, 2006GL026267. (10.1029/2006GL026267)

[10] Monerie PA, Robson J, Dong B, Hodson DLR, Klingaman NP. 2019 Effect of the atlantic multidecadal variability on the global monsoon. Geophys. Res. Lett. **46**, 1765-1775. (10.1029/2018GL080903)

[11] Omrani NE, Keenlyside NS, Bader J, Manzini E. 2014 Stratosphere key for wintertime atmospheric response to warm Atlantic decadal conditions. Clim. Dyn. **42**, 649-663. (10.1007/s00382-013-1860-3)

[12] Peings Y, Magnusdottir G. 2014 Forcing of the wintertime atmospheric circulation by the multidecadal fluctuations of the North Atlantic Ocean. Environ. Res. Lett. **9**, 034018. (10.1088/1748-9326/9/3/034018)

[13] Ruggieri P *et al.* 2021 Atlantic multidecadal variability and north atlantic jet: a multimodel view from the decadal climate prediction project. J. Climate **34**, 347-360. (10.1175/JCLI-D-19-0981.1)

[14] Folland CK, Knight J, Linderholm HW, Fereday D, Ineson S, Hurrell JW. 2009 The summer north atlantic oscillation: past, present, and future. J. Climate **22**, 1082-1103. (10.1175/2008JCLI2459.1)

[15] Dong B, Sutton RT, Woollings T, Hodges K. 2013 Variability of the North Atlantic summer storm track: mechanisms and impacts on European climate. Environ. Res. Lett. **8**, 034037. (10.1088/1748-9326/8/3/034037)

[16] Kucharski F, Kang IS, Farneti R, Feudale L. 2011 Tropical pacific response to 20th century Atlantic warming. Geophys. Res. Lett. **38**, 2010GL046248. (10.1029/2010GL046248)

[17] Ruprich-Robert Y *et al.* 2021 Impacts of Atlantic multidecadal variability on the tropical Pacific: a multi-model study. npj Climate Atmos. Sci. **4**, 33. (10.1038/s41612-021-00188-5)

[18] McGregor S, Timmermann A, Stuecker MF, England MH, Merrifield M, Jin FF, Chikamoto Y. 2014 Recent walker circulation strengthening and Pacific cooling amplified by Atlantic warming. Nat. Clim. Change **4**, 888-892. (10.1038/nclimate2330)

[19] Zhang R, Sutton R, Danabasoglu G, Kwon YO, Marsh R, Yeager SG, Amrhein DE, Little CM. 2019 A review of the role of the atlantic meridional overturning circulation in atlantic multidecadal variability and associated climate impacts. Rev. Geophys. **57**, 316-375. (10.1029/2019RG000644)

[20] Drinkwater KF, Miles M, Medhaug I, Otterå OH, Kristiansen T, Sundby S, Gao Y. 2014 The Atlantic multidecadal oscillation: its manifestations and impacts with special emphasis on the Atlantic region north of $60^{\circ }N$. J. Mar. Sys. **133**, 117-130. (10.1016/j.jmarsys.2013.11.001)

[21] Faillettaz R, Beaugrand G, Goberville E, Kirby RR. 2019 Atlantic Multidecadal Oscillations drive the basin-scale distribution of Atlantic bluefin tuna. Sci. Adv. **5**, eaar6993. (10.1126/sciadv.aar6993) 30613764 PMC6314829

[22] Straneo F, Heimbach P. 2013 North Atlantic warming and the retreat of Greenland’s outlet glaciers. Nature **504**, 36-43. (10.1038/nature12854) 24305146

[23] Tung KK, Zhou J. 2013 Using data to attribute episodes of warming and cooling in instrumental records. Proc. Natl Acad. Sci. USA **110**, 2058-2063. (10.1073/pnas.1212471110) 23345448 PMC3568361

[24] Mann ME, Steinman BA, Brouillette DJ, Miller SK. 2021 Multidecadal climate oscillations during the past millennium driven by volcanic forcing. Science **371**, 1014-1019. (10.1126/science.abc5810) 33674487

[25] Yan X, Zhang R, Knutson TR. 2018 Underestimated AMOC variability and implications for AMV and pedictability in CMIP models. Geophys. Res. Lett. **45**, 4319-4328. (10.1029/2018GL077378)

[26] Yan X, Zhang R, Knutson TR. 2019 A multivariate AMV index and associated discrepancies between observed and CMIP5 externally forced AMV. Geophys. Res. Lett. **46**, 4421-4431. (10.1029/2019GL082787)

[27] Robson J *et al.* 2018 Recent multivariate changes in the North Atlantic climate system, with a focus on 2005–2016. Int. J. Climatol. **38**, 5050-5076. (10.1002/joc.5815)

[28] Robson J, Sutton R, Lohmann K, Smith D, Palmer MD. 2012 Causes of the rapid warming of the north atlantic ocean in the mid-1990s. J. Climate **25**, 4116-4134. (10.1175/JCLI-D-11-00443.1)

[29] Zhang R. 2017 On the persistence and coherence of subpolar sea surface temperature and salinity anomalies associated with the Atlantic multidecadal variability. Geophys. Res. Lett. **44**, 7865-7875. (10.1002/2017GL074342)

[30] Gulev SK, Latif M, Keenlyside N, Park W, Koltermann KP. 2013 North Atlantic Ocean control on surface heat flux on multidecadal timescales. Nature **499**, 464-467. (10.1038/nature12268) 23887431

[31] O’Reilly CH, Huber M, Woollings T, Zanna L. 2016 The signature of low-frequency oceanic forcing in the Atlantic Multidecadal Oscillation. Geophys. Res. Lett. **43**, 2810-2818. (10.1002/2016GL067925)

[32] Thornalley DJ *et al.* 2018 Anomalously weak Labrador Sea convection and Atlantic overturning during the past 150 years. Nature **556**, 227-230. (10.1038/s41586-018-0007-4) 29643484

[33] McCarthy GD, Haigh ID, Hirschi JJM, Grist JP, Smeed DA. 2015 Ocean impact on decadal Atlantic climate variability revealed by sea-level observations. Nature **521**, 508-510. (10.1038/nature14491) 26017453

[34] Caesar L, Rahmstorf S, Robinson A, Feulner G, Saba V. 2018 Observed fingerprint of a weakening Atlantic Ocean overturning circulation. Nature **556**, 191-196. (10.1038/s41586-018-0006-5) 29643485

[35] Caesar L, McCarthy G, Thornalley D, Cahill N, Rahmstorf S. 2021 Current Atlantic meridional overturning circulation weakest in last millennium. Nat. Geosci. **14**, 118-120. (10.1038/s41561-021-00699-z)

[36] Buckley MW, Marshall J. 2016 Observations, inferences, and mechanisms of the Atlantic Meridional Overturning Circulation: a review. Rev. Geophys. **54**, 5-63. (10.1002/2015RG000493)

[37] Delworth T, Manabe S, Stouffer R. 1993 Interdecadal variations of the thermohaline circulation in a coupled ocean–atmosphere model. J. Climate **6**, 1993-2011. (10.1175/1520-0442(1993)006<1993:IVOTTC>2.0.CO;2)

[38] Knight JR, Allan RJ, Folland CK, Vellinga M, Mann ME. 2005 A signature of persistent natural thermohaline circulation cycles in observed climate. Geophys. Res. Lett. **32**, 2005GL024233. (10.1029/2005GL024233)

[39] Jungclaus JH, Haak H, Latif M, Mikolajewicz U. 2005 Arctic–North atlantic interactions and multidecadal variability of the meridional overturning circulation. J. Climate **18**, 4013-4031. (10.1175/JCLI3462.1)

[40] Sévellec F, Fedorov AV. 2013 The leading, interdecadal eigenmode of the atlantic meridional overturning circulation in a realistic ocean model. J. Climate **26**, 2160-2183. (10.1175/JCLI-D-11-00023.1)

[41] Ba J *et al.* 2014 A multi-model comparison of Atlantic multidecadal variability. Clim. Dyn. **43**, 2333-2348. (10.1007/s00382-014-2056-1)

[42] Wills RCJ, Armour KC, Battisti DS, Hartmann DL. 2019 Ocean–atmosphere dynamical coupling fundamental to the atlantic multidecadal oscillation. J. Climate **32**, 251-272. (10.1175/JCLI-D-18-0269.1)

[43] Lai WKM, Robson JI, Wilcox LJ, Dunstone N. 2022 Mechanisms of internal atlantic multidecadal variability in HadGEM3-GC3.1 at two different resolutions. J. Climate **35**, 1365-1383. (10.1175/JCLI-D-21-0281.1)

[44] Marshall J *et al.* 2001 North Atlantic climate variability: phenomena, impacts and mechanisms. Int. J. Climatol. **21**, 1863-1898. (10.1002/joc.693)

[45] Yeager S, Danabasoglu G. 2014 The origins of late-twentieth-century variations in the large-scale north atlantic circulation. J. Climate **27**, 3222-3247. (10.1175/JCLI-D-13-00125.1)

[46] Danabasoglu G, Landrum L, Yeager SG, Gent PR. 2019 Robust and nonrobust aspects of atlantic meridional overturning circulation variability and mechanisms in the community earth system model. J. Climate **32**, 7349-7368. (10.1175/JCLI-D-19-0026.1)

[47] Jackson LC, Biastoch A, Buckley MW, Desbruyères DG, Frajka-Williams E, Moat B, Robson J. 2022 The evolution of the North Atlantic Meridional Overturning Circulation since 1980. Nat. Rev. Earth Environ. **3**, 241-254. (10.1038/s43017-022-00263-2)

[48] Robson J, Ortega P, Sutton R. 2016 A reversal of climatic trends in the North Atlantic since 2005. Nat. Geosci. **9**, 513-517. (10.1038/ngeo2727)

[49] Bellomo K, Meccia VL, D’Agostino R, Fabiano F, Larson SM, von Hardenberg J, Corti S. 2023 Impacts of a weakened AMOC on precipitation over the Euro-Atlantic region in the EC-Earth3 climate model. Clim. Dyn. **61**, 3397-3416. (10.1007/s00382-023-06754-2)

[50] Jiang W, Gastineau G, Codron F. 2021 Multicentennial variability driven by salinity exchanges between the atlantic and the arctic ocean in a coupled climate model. J. Adv. Model. Earth Syst. **13**, e2020MS002366. (10.1029/2020MS002366)

[51] Meccia VL, Fuentes-Franco R, Davini P, Bellomo K, Fabiano F, Yang S, von Hardenberg J. 2023 Internal multi-centennial variability of the Atlantic Meridional Overturning Circulation simulated by EC-Earth3. Clim. Dyn. **60**, 3695-3712. (10.1007/s00382-022-06534-4)

[52] Menary MB, Park W, Lohmann K, Vellinga M, Palmer MD, Latif M, Jungclaus JH. 2012 A multimodel comparison of centennial Atlantic meridional overturning circulation variability. Clim. Dyn. **38**, 2377-2388. (10.1007/s00382-011-1172-4)

[53] Yamamoto A, Tatebe H, Nonaka M. 2020 On the emergence of the atlantic multidecadal SST signal: a key role of the mixed layer depth variability driven by north atlantic oscillation.J. Climate **33**, 3511-3531. (10.1175/JCLI-D-19-0283.1)

[54] Martin ER, Thorncroft C, Booth BBB. 2014 The multidecadal atlantic SST–sahel rainfall teleconnection in CMIP5 simulations. J. Climate **27**, 784-806. (10.1175/JCLI-D-13-00242.1)

[55] Clement A, Bellomo K, Murphy LN, Cane MA, Mauritsen T, Rädel G, Stevens B. 2015 The Atlantic Multidecadal Oscillation without a role for ocean circulation. Science **350**, 320-324. (10.1126/science.aab3980) 26472908

[56] Delworth TL, Zeng F, Zhang L, Zhang R, Vecchi GA, Yang X. 2017 The central role of ocean dynamics in connecting the north atlantic oscillation to the extratropical component of the atlantic multidecadal oscillation. J. Climate **30**, 3789-3805. (10.1175/JCLI-D-16-0358.1)

[57] Brown PT, Lozier MS, Zhang R, Li W. 2016 The necessity of cloud feedback for a basin-scale Atlantic Multidecadal Oscillation. Geophys. Res. Lett. **43**, 3955-3963. (10.1002/2016GL068303)

[58] Bellomo K, Clement AC, Murphy LN, Polvani LM, Cane MA. 2016 New observational evidence for a positive cloud feedback that amplifies the Atlantic Multidecadal Oscillation. Geophys. Res. Lett. **43**, 9852-9859. (10.1002/2016GL069961)

[59] Yuan T, Oreopoulos L, Zelinka M, Yu H, Norris JR, Chin M, Platnick S, Meyer K. 2016 Positive low cloud and dust feedbacks amplify tropical North Atlantic Multidecadal Oscillation. Geophys. Res. Lett. **43**, 1349-1356. (10.1002/2016GL067679) 32818003 PMC7430503

[60] Qasmi S, Cassou C, Boé J. 2017 Teleconnection between atlantic multidecadal variability and european temperature: diversity and evaluation of the coupled model intercomparison project phase 5 models. Geophys. Res. Lett. **44**, 11 140-11 149. (10.1002/2017GL074886)

[61] Baek SH, Kushnir Y, Robinson WA, Lora JM, Lee DE, Ting M. 2021 An atmospheric bridge between the subpolar and tropical atlantic regions: a perplexing asymmetric teleconnection. Geophys. Res. Lett. **48**, e2021GL096602. (10.1029/2021GL096602)

[62] Booth BB, Dunstone NJ, Halloran PR, Andrews T, Bellouin N. 2012 Aerosols implicated as a prime driver of twentieth-century North Atlantic climate variability. Nature **484**, 228-232. (10.1038/nature10946) 22498628

[63] Bellomo K, Murphy LN, Cane MA, Clement AC, Polvani LM. 2018 Historical forcings as main drivers of the Atlantic multidecadal variability in the CESM large ensemble. Clim. Dyn. **50**, 3687-3698. (10.1007/s00382-017-3834-3)

[64] Murphy LN, Bellomo K, Cane M, Clement A. 2017 The role of historical forcings in simulating the observed Atlantic multidecadal oscillation. Geophys. Res. Lett. **44**, 2472-2480. (10.1002/2016GL071337)

[65] Otterå OH, Bentsen M, Drange H, Suo L. 2010 External forcing as a metronome for Atlantic multidecadal variability. Nat. Geosci. **3**, 688-694. (10.1038/ngeo955)

[66] Menary MB, Scaife AA. 2014 Naturally forced multidecadal variability of the Atlantic meridional overturning circulation. Clim. Dyn. **42**, 1347-1362. (10.1007/s00382-013-2028-x)

[67] Swingedouw D, Ortega P, Mignot J, Guilyardi E, Masson-Delmotte V, Butler PG, Khodri M, Séférian R. 2015 Bidecadal North Atlantic ocean circulation variability controlled by timing of volcanic eruptions. Nat. Commun. **6**, 1-12. (10.1038/ncomms7545) 25818017

[68] Iwi AM, Hermanson L, Haines K, Sutton RT. 2012 Mechanisms linking volcanic aerosols to the atlantic meridional overturning circulation. J. Climate **25**, 3039-3051. (10.1175/2011JCLI4067.1)

[69] Bellucci A, Mariotti A, Gualdi S. 2017 The role of forcings in the twentieth-century north atlantic multidecadal variability: the 1940–75 north atlantic cooling case study. J. Climate **30**, 7317-7337. (10.1175/JCLI-D-16-0301.1)

[70] Watanabe M, Tatebe H. 2019 Reconciling roles of sulphate aerosol forcing and internal variability in Atlantic multidecadal climate changes. Clim. Dyn. **53**, 4651-4665. (10.1007/s00382-019-04811-3)

[71] Murphy LN, Klavans JM, Clement AC, Cane MA. 2021 Investigating the roles of external forcing and ocean circulation on the atlantic multidecadal sst variability in a large ensemble climate model hierarchy. J. Climate **34**, 4835-4849. (10.1175/JCLI-D-20-0167.1)

[72] Klavans JM, Clement AC, Cane MA, Murphy LN. 2022 The evolving role of external forcing in north atlantic SST variability over the last millennium. J. Climate **35**, 2741-2754. (10.1175/JCLI-D-21-0338.1)

[73] Wilcox LJ, Highwood EJ, Dunstone NJ. 2013 The influence of anthropogenic aerosol on multi-decadal variations of historical global climate. Environ. Res. Lett. **8**, 024033. (10.1088/1748-9326/8/2/024033)

[74] Undorf S, Bollasina MA, Hegerl GC. 2018 Impacts of the 1900–74 increase in anthropogenic aerosol emissions from north america and europe on eurasian summer climate. J. Climate **31**, 8381-8399. (10.1175/JCLI-D-17-0850.1)

[75] Fiedler S, Putrasahan D. 2021 How does the north atlantic SST pattern respond to anthropogenic aerosols in the 1970s and 2000s? Geophys. Res. Lett. **48**, e2020GL092142. (10.1029/2020GL092142)

[76] Menary MB, Roberts CD, Palmer MD, Halloran PR, Jackson L, Wood RA, Müller WA, Matei D, Lee SK. 2013 Mechanisms of aerosol-forced AMOC variability in a state of the art climate model. J. Geophys. Res.: Oceans **118**, 2087-2096. (10.1002/jgrc.20178)

[77] Undorf S, Bollasina MA, Booth BBB, Hegerl GC. 2018 Contrasting the effects of the 1850–1975 increase in sulphate aerosols from north america and europe on the Atlantic in the CESM. Geophys. Res. Lett. **45**, 11 930-11 940. (10.1029/2018GL079970)

[78] Menary MB *et al.* 2020 Aerosol-forced AMOC Changes in CMIP6 historical simulations. Geophys. Res. Lett. **47**, e2020GL088166. (10.1029/2020GL088166)

[79] Robson J, Menary MB, Sutton RT, Mecking J, Gregory JM, Jones C, Sinha B, Stevens DP, Wilcox LJ. 2022 The role of anthropogenic aerosol forcing in the 1850–1985 strengthening of the AMOC in CMIP6 historical simulations. J. Climate **35**, 3243-3263. (10.1175/JCLI-D-22-0124.1)

[80] Andrews MB *et al.* 2020 Historical simulations with HadGEM3-GC3.1 for CMIP6. J. Adv. Model. Earth Syst. **12**, e2019MS001995. (10.1029/2019MS001995)

[81] Baek SH, Kushnir Y, Ting M, Smerdon JE, Lora JM. 2022 Regional signatures of forced north atlantic SST variability: a limited role for aerosols and greenhouse gases. Geophys. Res. Lett. **49**, e2022GL097794. (10.1029/2022GL097794)

[82] Zhang R *et al.* 2013 Have aerosols caused the observed atlantic multidecadal variability? J. Atmos. Sci. **70**, 1135-1144. (10.1175/JAS-D-12-0331.1)

[83] Kim WM, Yeager SG, Danabasoglu G. 2018 Key role of internal ocean dynamics in atlantic multidecadal variability during the last half century. Geophys. Res. Lett. **45**, 13 449-13 457. (10.1029/2018GL080474)

[84] Zelinka MD, Andrews T, Forster PM, Taylor KE. 2014 Quantifying components of aerosol-cloud-radiation interactions in climate models. J. Geophys. Res.: Atmos. **119**, 7599-7615. (10.1002/2014JD021710)

[85] Smith CJ *et al.* 2021 Energy budget constraints on the time history of aerosol forcing and climate sensitivity. J. Geophys. Res.: Atmos. **126**, e2020JD033622. (10.1029/2020JD033622)

[86] Flynn CM, Mauritsen T. 2020 On the climate sensitivity and historical warming evolution in recent coupled model ensembles. Atmos. Chem. Phys. **20**, 7829-7842. (10.5194/acp-20-7829-2020)

[87] Wang C, Soden BJ, Yang W, Vecchi GA. 2021 Compensation between cloud feedback and aerosol-cloud interaction in CMIP6 models. Geophys. Res. Lett. **48**, e2020GL091024. (10.1029/2020GL091024)

[88] Zhang J *et al.* 2021 The role of anthropogenic aerosols in the anomalous cooling from 1960 to 1990 in the CMIP6 Earth system models. Atmos. Chem. Phys. **21**, 18 609-18 627. (10.5194/acp-21-18609-2021)

[89] Sutton RT, Dong B, Gregory JM. 2007 Land/sea warming ratio in response to climate change: IPCC AR4 model results and comparison with observations. Geophys. Res. Lett. **34**, 2006GL028164. (10.1029/2006GL028164)

[90] Trenberth KE, Shea DJ. 2006 Atlantic hurricanes and natural variability in 2005. Geophys. Res. Lett. **33**, 2006GL026894. (10.1029/2006GL026894)

[91] Deser C, Phillips AS. 2021 Defining the internal component of atlantic multidecadal variability in a changing climate. Geophys. Res. Lett. **48**, e2021GL095023. (10.1029/2021GL095023)

[92] Deser C, Phillips AS. 2023 Spurious indo-pacific connections to internal atlantic multidecadal variability introduced by the global temperature residual method. Geophys. Res. Lett. **50**, e2022GL100574. (10.1029/2022GL100574)

[93] Monerie PA, Sanchez-Gomez E, Pohl B, Robson J, Dong B. 2017 Impact of internal variability on projections of Sahel precipitation change. Environ. Res. Lett. **12**, 114003. (10.1088/1748-9326/aa8cda)

[94] Eyring V, Bony S, Meehl GA, Senior CA, Stevens B, Stouffer RJ, Taylor KE. 2016 Overview of the coupled model intercomparison project phase 6 (CMIP6) experimental design and organization. Geosci. Model Dev. **9**, 1937-1958. (10.5194/gmd-9-1937-2016)

[95] Huang B *et al.* 2017 Extended reconstructed sea surface temperature, version 5 (ERSSTv5): upgrades, validations, and intercomparisons. J. Climate **30**, 8179-8205. (10.1175/JCLI-D-16-0836.1)

[96] Tandon NF, Kushner PJ. 2015 Does external forcing interfere with the AMOC’s influence on north atlantic sea surface temperature? J. Climate **28**, 6309-6323. (10.1175/JCLI-D-14-00664.1)

[97] Bellucci A, Mattei D, Ruggieri P, Famooss Paolini L. 2022 Intermittent behavior in the AMOC-AMV relationship. Geophys. Res. Lett. **49**, e2022GL098771. (10.1029/2022GL098771)

[98] Terray L. 2012 Evidence for multiple drivers of North Atlantic multi-decadal climate variability. Geophys. Res. Lett. **39**, 2012GL053046. (10.1029/2012GL053046)

[99] Menary MB, Hodson DLR, Robson JI, Sutton RT, Wood RA, Hunt JA. 2015 Exploring the impact of CMIP5 model biases on the simulation of North Atlantic decadal variability. Geophys. Res. Lett. **42**, 5926-5934. (10.1002/2015GL064360)

[100] Weijer W, Cheng W, Garuba OA, Hu A, Nadiga BT. 2020 CMIP6 models predict significant 21st century decline of the atlantic meridional overturning circulation. Geophys. Res. Lett. **47**, e2019GL086075. (10.1029/2019GL086075)

[101] Scaife AA, Smith D. 2018 A signal-to-noise paradox in climate science. npj Climate Atmos. Sci. **1**, 1-8. (10.1038/s41612-018-0038-4)

[102] Kim WM, Yeager S, Chang P, Danabasoglu G. 2018 Low-frequency north atlantic climate variability in the community earth system model large ensemble. J. Climate 31, 787-813. (10.1175/JCLI-D-17-0193.1)

[103] Smith DM *et al.* 2022 Attribution of multi-annual to decadal changes in the climate system: the large ensemble single forcing model intercomparison project (LESFMIP). Front. Climate **4**, 955414. (10.3389/fclim.2022.955414)

[104] Robson J, Sutton R, Menary MB, Lai MWK. 2023 Contrasting internally and externally generated Atlantic Multidecadal Variability and the role for AMOC in CMIP6 historical simulations. Figshare. (10.6084/m9.figshare.c.6824114) PMC10590668 37866382

